# Supplementary material for: Diversity of CFTR variants across ancestries characterized using 454,727 UK biobank whole exome sequences
Source: Genome Med. 2024 Mar 21;16:43. doi: 10.1186/s13073-024-01316-5 (PMC10956269; doi:10.1186/s13073-024-01316-5)
Supplement: Supplementary file 2 — Additional file 2: Fig. S1. Venn diagram showing genealogical estimation of variant age (GEVA) analysis of CF-causing variants. [file 13073_2024_1316_MOESM2_ESM.pptx]

## Slide 1
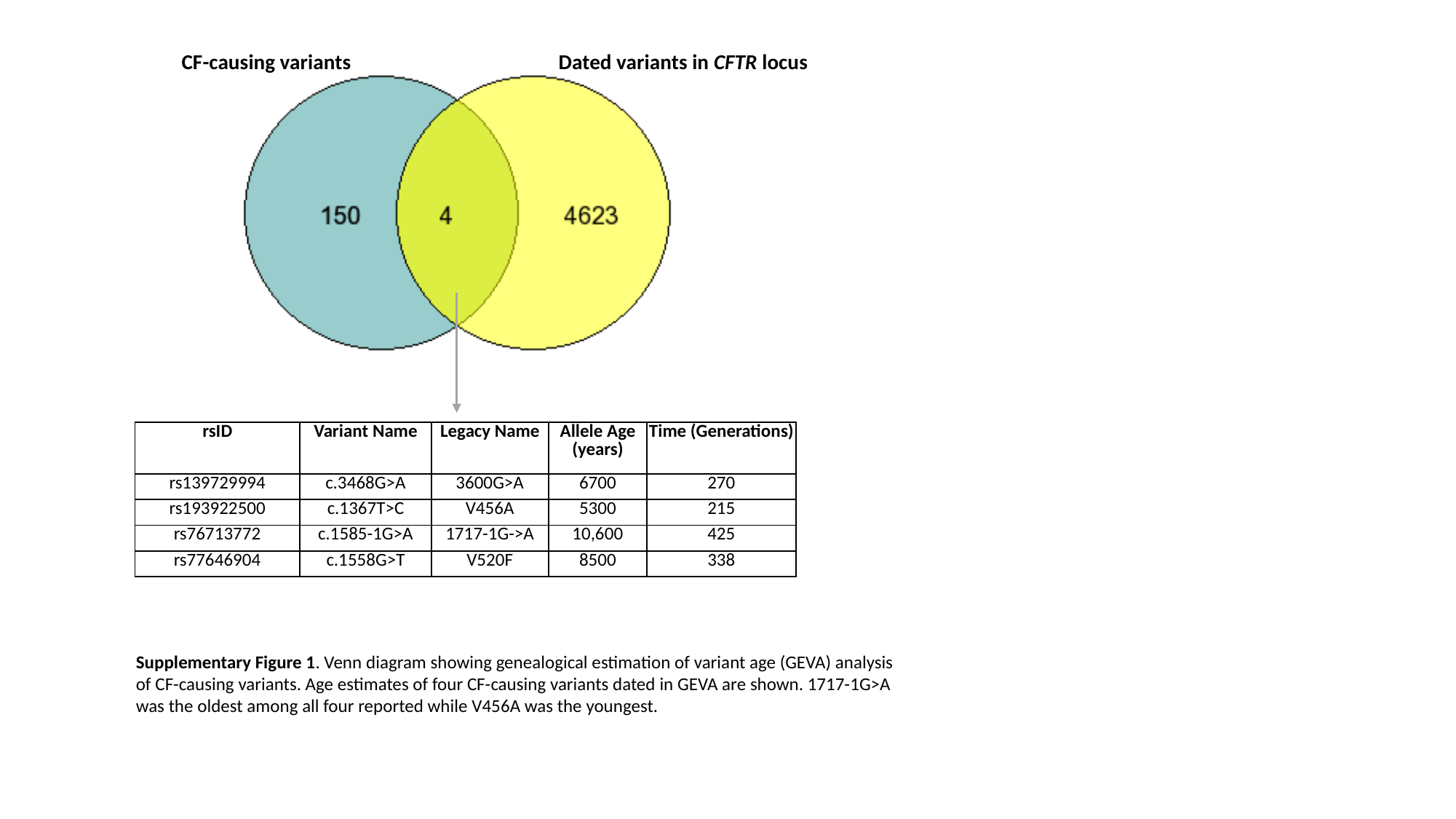

CF-causing variants
Dated variants in CFTR locus
| rsID | Variant Name | Legacy Name | Allele Age (years) | Time (Generations) |
| --- | --- | --- | --- | --- |
| rs139729994 | c.3468G>A | 3600G>A | 6700 | 270 |
| rs193922500 | c.1367T>C | V456A | 5300 | 215 |
| rs76713772 | c.1585-1G>A | 1717-1G->A | 10,600 | 425 |
| rs77646904 | c.1558G>T | V520F | 8500 | 338 |
Supplementary Figure 1. Venn diagram showing genealogical estimation of variant age (GEVA) analysis of CF-causing variants. Age estimates of four CF-causing variants dated in GEVA are shown. 1717-1G>A was the oldest among all four reported while V456A was the youngest.
